# Multiple Factors Involved in Nonalcoholic Hepatitis Pathogenesis

**DOI:** 10.1155/2012/429805

**Published:** 2012-12-20

**Authors:** Manuela Neuman, Nir Hilzenrat, Lawrence Cohen, Robert E. Winkler, Radu Nanau

**Affiliations:** ^1^Departments of Clinical Pharmacology and Toxicology, and Global Health, University of Toronto, Toronto, ON, Canada M5G 1L7; ^2^In Vitro Drug Safety and Biotechnology, University of Toronto, MaRS Discovery District, 101 College Street, Suite 300, Lab 351, Toronto, ON, Canada M5G 1L7; ^3^Division of Gastroenterology, Jewish General Hospital and McGill University, Montreal, QC, Canada H9H 3L1; ^4^Division of Gastroenterology, Sunnybrook HSC, University of Toronto, Toronto, ON, Canada M4N 3M5; ^5^Amicus Pharmaceuticals, Cranbury, NJ 08512, USA

Nonalcoholic fatty liver disease (NAFLD) is the most common type of liver disease, with etiologies as varied as its presentations. This special issue of the International Journal of Hepatology examines some of the multiple factors involved in the pathogenesis of this chronic liver condition, ranging from inflammation, aberrant lipid metabolism, drug-induced liver injury, and babesiosis.

NAFLD is marked by a high degree of inflammation. K. Tajiri and Y. Shimizu (2012) describe the role of natural killer T (NKT) cells in NAFLD pathogenesis. The role of these inflammatory cells changes throughout the course of the disease, progressing from being initially protective during steatosis, to acting as progression factors during fibrosis. The interaction between NKT cells and the glycolipid antigen-presenting CD1d molecule is believed to play a key role in this process. NKT cells are lipid antigen-specific lymphocytes that produce high levels of T helper 1 response proinflammatory cytokines. NKT cells also have anti-inflammatory properties through T helper 2 polarization, thus playing a key role in modulating NAFLD. CD1d was shown to lead to hepatic inflammation through antigen presentation to NKT cells. In turn, this process is associated with insulin resistance and altered lipid metabolism. Thus, manipulating NKT cells may provide a therapeutic avenue in NAFLD (K. Tajiri and Y. Shimizu, 2012).

In the “two-hit theory” model, fatty acids and triglycerides accumulate in the liver, leading to inflammation, oxidative stress, and mitochondrial dysfunction, ultimately causing liver damage. M. Enjoji et al. (2012) review this mechanism from the point of view of cholesterol metabolism, while discussing its management as a potential treatment for NAFLD. NAFLD is marked by dysfunctional cholesterol metabolism, with cholesterol accumulation taking place as a result of *de novo* synthesis and plateauing excretion observed even if dietary intake is high. Based on findings from a Japanese population, the highest cholesterol intake was observed in nonobese NAFLD patients, while obese NAFLD patients had higher cholesterol intake than healthy volunteers. Excess liver cholesterol and its metabolite oxysterol are associated with steatosis and activation of the liver X receptor-*α*-sterol regulatory element-binding protein-1c pathway. Inhibiting cholesterol absorption or reducing dietary cholesterol intake may help reduce hepatocytic cholesterol accumulation, thus presenting valid cholesterol management techniques that may aid in the prevention of cholesterol overload-associated NAFLD (M. Enjoji et al., 2012).

Diets of obese patients are generally high in fat, particularly saturated fats and Ω-6 polyunsaturated fatty acids. Obesity is marked by a high volume of adipose tissue, which is associated with increased production of monocyte chemoattractant protein (MCP-) 1. In turn, MCP-1 attracts macrophages, which leads to the development of an inflammatory cascade associated with high levels of proinflammatory cytokines, among which tumor necrosis factor-*α* is associated with insulin resistance and impaired glucose tolerance (P. Guturu and A. Duchini, 2012). Free fatty acids accumulate in the liver when a greater amount is delivered to the organ than that which is metabolized, particularly when coupled with deregulated *de novo* synthesis. P. Guturu and A. Duchini (2012) further argue that the type of free fatty acids is more important than their quantity with respect to the development and progression of NAFLD. Of particular concern were saturated fatty acids.

Paraoxonase (PON) is an esterase associated with the hydrolysis of various xenobiotics. Serum PON1 is synthesized in the liver. Serum PON1 levels are low in nonalcoholic steatohepatitis (NASH) patients. Furthermore, PON can be inactivated by oxidative stress, while decreased antioxidative potential may facilitate the evolution of NAFLD to NASH. *In vitro* experiments have shown that proinflammatory cytokines decrease PON1 mRNA levels, while decreased PON1 activity is related to the degree of liver injury in patients (O. Hussein et al., 2012). A methionine choline deficient diet (MCDD) was associated with increased liver weight in rats, characterized by substantial increases in hepatic triglycerides and cholesterol levels. Furthermore, MCDD is associated with decreased PON activity, as well as increased malondialdehyde levels (indicator of oxidation) in both serum and liver. Serum activity of PON was increased when animals were treated with metformin (M), rosiglitazone (R), ezetimibe (E), valsartan (V), M + R, R + M + V or R + M + V + E. In contrast, liver PON activity was only increased in animals treated with R, E, V, R + M + V and R + M + V + E. O. Hussein et al. (2012) show that insulin sensitizers decrease oxidative stress in both serum and liver. 

The management of human immunodeficiency virus (HIV) infection is dependent upon the efficacy and safety of the different highly active antiretroviral therapy (HAART) regimens used. While hepatotoxicity is often present in HIV patients and generally improves upon HAART initiation, the intrinsic ability of certain antiretrovirals to cause hepatotoxicity is a factor that limits their usefulness. Hepatotoxicity is recognized as main type of adverse drug reaction (ADR) associated with HAART. Baseline hepatotoxicity, alcohol consumption, preexisting viral hepatitis, and old age are risk factors for developing drug-induced hepatotoxicity in HIV-positive patients (M. Neuman et al., 2012). Hepatotoxicity can manifest itself as hepatocellular injury, cholestasis, a mixed pattern of cytotoxic and cholestatic injury, or, less commonly, steatosis. Another common type of ADR affecting the liver is hypersensitivity reaction (HSR). Furthermore, gastrointestinal intolerance and pancreatitis are recognized as other treatment-limiting ADRs associated with HAART. The management of these reactions is paramount as they can lead to treatment non-adherence, which in turn can give rise to viral resistance. The authors conclude that therapeutic and drug monitoring of ART, using laboratory identification of phenotypic susceptibilities, drug interactions with other medications, drug interactions with herbal medicines, and alcohol intake might enable a safer use of this medication (M. Neuman et al., 2012). 

J. C. Pritchett et al. (2012) describe in detail the association between drug-induced hypersensitivity reaction (DIHS), with a particular focus on both cutaneous and hepatic symptoms, and the reactivation of latent human herpes virus (HHV-) 6 infection. A “true” HSR is described by the triad of rash, fever, and organ involvement. The internal organ most often affected in the liver. Drug-induced liver injury presents itself as anomalies in liver function tests or hepatomegaly. Cutaneous reactions often present as maculopapular rash or generalized erythematous rash. HHV-6 is a lymphotropic DNA virus infecting close to 100% of the population in the first 2-3 years of life. Following the initial infection, HHV-6 remains dormant in the body and can become reactivated under conditions of immunosuppression (J. C. Pritchett et al., 2012). DIHS cases associated with HHV-6 reactivation show a more severe set of symptoms than cases without viral reactivation. Common symptoms of DIHS include fever, edema, lymphadenopathy, hypereosinophilia, and atypical lymphocytes, as well as elevated serum aminotransferase levels, cholestatic hepatitis, renal insufficiency, and multiorgan failure. Hypogammaglobulinemia is also widely observed, while elevations in serum IgG levels often indicate viral reactivation. Antiepileptics, sulfonamide antibiotics, allopurinol, and nonsteroidal anti-inflammatory drugs are some of the pharmaceutical agents associated with DIHS development. Symptoms of HSR generally improve following the discontinuation of the offending agent, but may relapse after 2-3 weeks, which coincides with the detection of viral reactivation (J. C. Pritchett et al., 2012). 

Human babesiosis is transmitted through tick (*Idoxes* sp.) bites or through transfusion of infected blood. Symptoms of babesiosis include hepatitis, hydrothorax, pneumonia, myocarditis, splenomegaly, glomerulonephritis, hematuria, and hemolytic anemia. Individuals most at risk of developing this condition are neonates and the elderly, as well as splenectomised, immunocompromised, and AIDS patients. If not adequately treated, babesiosis can be fatal (H. S. Oz and K. H. Westlund, 2012). Hamsters are the preferred animal model in which to study babesiosis development and drug treatment. Treatments include the clindamycin/quinine combination, azithromycin/quinine, and atovaquone. Symptoms of babesiosis include low hemoglobin, high bilirubin, and elevated aspartate aminotransferase, creatinine, and hemoglobinuria levels. H. S. Oz and K. H. Westlund (2012) advocate for increased awareness, as well as improved diagnostic and therapeutic tools. An important preventive tool would be to screen blood donors for infection.

*Manuela Neuman* *Manuela Neuman* *Robert E. Winkler* *Robert E. Winkler* *Nir Hilzenrat* *Nir Hilzenrat* *Lawrence Cohen* *Lawrence Cohen* *Radu M. Nanau* *Radu M. Nanau*

